# Structural basis of transcobalamin recognition by human CD320 receptor

**DOI:** 10.1038/ncomms12100

**Published:** 2016-07-14

**Authors:** Amer Alam, Jae-Sung Woo, Jennifer Schmitz, Bernadette Prinz, Katharina Root, Fan Chen, Joël S. Bloch, Renato Zenobi, Kaspar P. Locher

**Affiliations:** 1 Department of Biology, Institute of Molecular Biology and Biophysics, ETH Zurich, 8093 Zurich, Switzerland; 2 Department of Chemistry and Applied Biosciences, ETH Zurich, 8093 Zurich, Switzerland

## Abstract

Cellular uptake of vitamin B12 (cobalamin) requires capture of transcobalamin (TC) from the plasma by CD320, a ubiquitous cell surface receptor of the LDLR family. Here we present the crystal structure of human holo-TC in complex with the extracellular domain of CD320, visualizing the structural basis of the TC-CD320 interaction. The observed interaction chemistry can rationalize the high affinity of CD320 for TC and lack of haptocorrin binding. The *in vitro* affinity and complex stability of TC-CD320 were quantitated using a solid-phase binding assay and thermostability analysis. Stable complexes with TC were also observed for the disease-causing CD320ΔE88 mutant and for the isolated LDLR-A2 domain. We also determined the structure of the TC-CD320ΔE88 complex, which revealed only minor changes compared with the wild-type complex. Finally, we demonstrate significantly reduced *in vitro* affinity of TC for CD320 at low pH, recapitulating the proposed ligand release during the endocytic pathway.

## Abstract (web summary)

Cellular uptake of vitamin B12 (cobalamin) requires the binding of holo-transcobalamin (TC) from plasma by CD320. Here, the authors report the structure of a complex between CD320 and TC loaded with cyanocobalamin, alongside additional functional analysis.

## Graphical abstract

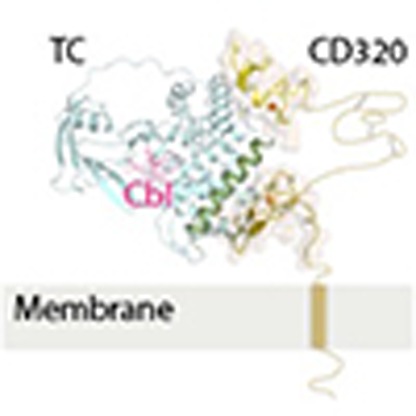

Mammals depend on dietary uptake of Cobalamin (Cbl), which is a cofactor of at least two enzymes that are part of essential biochemical pathways^1^,^2^. A complex uptake system involving several transport proteins and cellular receptors facilitates efficient transport of this scarce vitamin^3^,^4^,^5^,^6^,^7^,^8^,^9^,^10^. Defects at any stage of this process have been found to lead to Cbl deficiency and severe developmental defects in patients^11^,^12^. transcobalamin (TC)-bound Cbl is transported into cells by receptor-mediated endocytosis, which requires Ca^2+^-dependent complex formation of TC with its cognate cell surface receptor CD320 (ref. 13). CD320 is a member of the Low-density Lipoprotein Receptor (LDLR) family^13^,^14^, a large group of mostly multi-ligand receptors involved in cholesterol uptake and other physiologically essential functions^15^,^16^. CD320 features an N-terminal, extracellular fragment comprising two LDLR type A (LDLR-A) domains separated by an epidermal growth factor (EGF) homology domain, the exact function of which has so far not been determined. It also contains a single transmembrane helix and a C-terminal cytoplasmic region involved in complex internalization^8^. While the majority of related LDLR domain-containing receptors bind multiple and distinct ligands^17^, CD320 has been reported to bind TC with high specificity and affinity as evidenced by the lack of interference in TC binding from related LDLR ligands like LDL and RAP as well as high affinity for TC of both the full-length receptor and its soluble ectodomain^8^,^13^,^18^. Even haptocorrin (HC), a high affinity Cbl-binding protein that shares significant sequence and structural similarities with TC and is also circulating in the plasma, is not recognized by CD320.

Although several of the proteins facilitating Cbl uptake and transport from saliva to the blood stream have been structurally characterized^19^,^20^,^21^,^22^, the final uptake step into cells has not been visualized at a molecular level. Such insight is required not only to understand the basis of specific TC-Cbl uptake into cells, but also to rationalize disease-causing mutations that interfere with this process. Furthermore, expression of CD320 is reported to be highest in actively proliferating cells and is strongly upregulated in many cancer cells, prompting interest in its potential targeting through receptor directed drug delivery^23^,^24^ or its exploitation for tumour diagnostics. In light of its physiological relevance, we set out to biochemically and structurally characterize the TC-CD320 complex, revealing key molecular details of the high affinity interaction, its regulation by pH and the *in vitro* effect of mutations of CD320 on its interaction with TC.

## Results

### Structural and functional analysis of TC-CD320 complex

The challenges associated with the production of suitable amounts of homogenous, stable, and functional CD320 protein have so far prevented its structural analysis^7^,^25^,^26^. To overcome these challenges, we heterologously co-expressed human TC and the external, TC-binding part of CD320 in secreted form in baculovirus-infected insect cells, taking advantage of the enhanced biochemical stability of the complex over individual components. CD320 was N-terminally fused to maltose-binding protein (MBP) that was subsequently cleaved on purification. We purified the TC-CD320 complex to homogeneity in the presence of excess cyanocobalamin (Cbl-CN) to ensure full occupancy of the Cbl-binding site. The obtained complex had a stoichiometry of 1:1 as confirmed by MALDI mass spectrometry (Supplementary Fig. 1).

**Figure 1:**
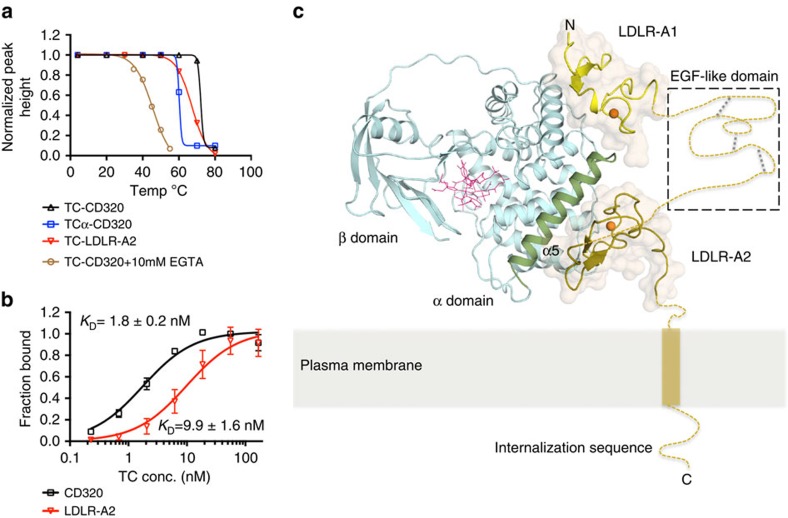
Characterization of the TC-CD320 complex. (**a**) Thermostability curves for different TC-CD320 complexes, demonstrating decreased complex stability in the presence of EGTA as well as for the TCα-CD320 and TC-LDLR-A2 complexes. (**b**) Solid-phase binding assay quantitating TC binding to full-length CD320 (black) and to LDLR-A2 (red; *n*=5, error bars indicate S.D). (**c**) Overall structure of TC-CD320 complex showing TC (cyan) and LDLR-A1 and LDLR-A2 of CD320 (yellow and gold, respectively). The likely position of the EGF-like module (boxed) bridging the two LDLR-A domains is shown schematically. The transmembrane domain (yellow cylinder) and internalization motif of CD320 were not part of the expression construct. Orange spheres represent bound Ca^2+^ ions. Bound Cbl, sandwiched between the α and β domains of TC, is shown in pink and TC helix α5 is coloured green.

The stability of the TC-CD320 complex was analysed by determining its thermostability upon incubation at increasing temperatures, followed by size exclusion chromatography (SEC) analysis. As shown in Fig. 1a, we observed a >20 °C drop in the apparent Tm in the presence of the Ca^2+^ chelator ethylene glycol tetraacetic acid (EGTA), confirming the stabilizing effect of Ca^2+^ on the TC-CD320 complex. We developed an enzyme-linked immunosorbent assay-based solid-phase binding assay to determine the affinity of the TC-CD320 interaction by using immobilized MBP-tagged, biotinylated CD320 (CD320_AVI_). Tag-less TC was obtained by EGTA induced dissociation of a pre-formed TC-CD320_AVI_ complex immobilized on streptavidin resin. TC binding was quantitated using a commercial monoclonal antibody (A-5) targeting a distinct C-terminal epitope of TC, away from its receptor-binding site. As shown in Fig. 1b, TC was confirmed to bind to CD320 with high affinity (*K*_D_∼1.5 nM), in line with the high thermal stability of the complex.

To analyse whether a single LDLR-A domain of CD320 was capable of binding TC (analogous to observations with the Reelin/LDLR-A1 complex^27^), we individually co-expressed each domain with TC. Complex formation was observed both for LDLR-A1 and LDLR-A2, which is in contrast to a previous suggestion that two LDLR-A modules are the minimal binding unit for TC (ref. 8). We found that a complex containing only LDLR-A2 had an apparent Tm only ∼5 °C lower than the equivalent complex containing full-length CD320, and a dissociation constant of ∼10 nM, slightly reduced compared to full-length CD320 (Fig. 1b). In contrast, the complex with LDLR-A1 appeared less stable on purification, indicating a decreased stability and precluding a detailed thermostability or solid-phase binding analysis.

Various CD320 truncations were generated and tested and a final crystallization construct comprising residues 53–199 of the extracellular domain of CD320 and TC residues 1–409 was chosen for structure determination. The TC-CD320 co-crystal structure, phased by molecular replacement using Cbl-bound TC as a search model, was refined to 2.1 Å resolution and revealed a 1:1 complex of TC-CD320 (Fig. 1c). Whereas clear electron density was observed for LDLR-A1 residues 53–89 and LDLR-A2 residues 129-171, no density was observed for the EGF-like domain (residues 90–128). Both LDLR-A domains and TC contain several disulfide bridges, the positions of the majority of which were confirmed using an anomalous difference map calculated from data collected at a wavelength of 1.6 Å (Table 1). In the same map, Ca^2+^ ions bound to the LDLR-A domains and the Cobalt of Cbl bound to TC were identified.

### Overall architecture of the TC-CD320 complex

In agreement with epitope mapping studies^28^, both LDLR-A1 and LDLR-A2 of CD320 exclusively interact with the α domain of TC (Fig. 1c). The structure of CD320-bound TC is very similar to that of its free state^20^ (r.m.s.d of all Cα atoms of 0.43Å, Supplementary Fig. 2) with a few key differences. First, we observed electron density for a fourth disulfide bridge linking residues Cys65-Cys78, both of which are conserved in several primate and rodent species^20^. Second, our structure revealed clear electron density for the loop connecting the α and β domains of TC. Finally, we observed electron density for the loop carrying the Cbl-binding His173.

To confirm the implications of the observed interface, we co-expressed CD320 with a truncated version of TC containing only its α domain (TCα) to mimic its apo form. We expressed the TCα–CD320 complex in insect cells to avoid denaturing conditions previously used to obtain apo-TC (ref. 29). We could indeed purify a TCα–CD320 complex with a 1:1 stoichiometry, which exhibited a decreased stability (apparent Tm of ∼60 °C) compared with the complex containing full-length TC (apparent Tm of ∼70 °C, Fig. 1a).

**Figure 2:**
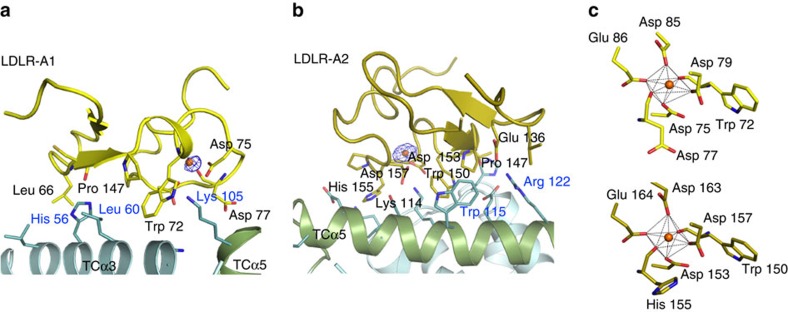
Details of the TC-CD320 binding interface. (**a**) Interactions of LDLR-A1 and (**b**) LDLR-A2 with TC. TC helix α5 is coloured green. Ca^2+^ ions are indicated as orange spheres with corresponding anomalous difference density peaks shown (blue mesh contoured at 4*σ*). TC and CD320 residues involved in intermolecular contacts and Ca^2+^ coordination are shown as sticks and labelled blue and black, respectively. (**c**) Close-up views showing octahedral Ca^2+^ coordination in LDLR-A1 (yellow, top) and LDLR-A2 (gold, bottom).

The interface of TC and CD320 consists of ∼900 Å^2^ buried surface area, with LDLR-A2 contributing 486 Å^2^ compared with 405 Å^2^ for LDLR-A1. The fold of the LDLR-A domains of CD320 is similar to that of related family members^30^,^31^,^32^,^33^,^34^ (Fig. 2a–c). Both LDLR-A domains contain central Ca^2+^ ions bound by four conserved acidic residues and two backbone carbonyls in an octahedral coordination. They also contain a PLxWRCD motif that is conserved in other CD320 orthologs and related proteins^13^. The backbone carbonyl of the tryptophan residue in this motif (Trp72 and Trp150 in LDLR-A1 and LDLR-A2, respectively) coordinates the central Ca^2+^ ion, while its side chain is stabilized through a stacking interaction with the preceding proline residue. In the case of LDLR-A2, this interaction is further stabilized through stacking with Trp115 and hydrogen bonding with Glu118 of TC. These latter interactions are absent in the LDLR-A1/TC interface. In LDLR-A1, the Ca^2+^-coordinating carboxylate groups of Asp75 and Asp79 form salt bridges with Lys105 of TC, forming an ‘acidic necklace' previously found to be a key feature of LDLR/ligand complexes^32^ (Fig. 2a). The equivalent acidic necklace in LDLR-A2 is formed by Lys114 of TC and the Ca^2+^ coordinating carboxylate groups of Asp153 and Asp157 of CD320 (Fig. 2b).

A network of disulfide linkages stabilizes the folds of both LDLR-A1 and LDLR-A2, with disulfide formation following the 1-3/2-5/4-6 pattern observed previously^30^,^31^,^32^,^34^,^35^. LDLR-A1 contacts TC helices α3 and α5, whereas LDLR-A2 interacts with TC helix α5 and the loop between helices α6 and α7 (Fig. 2b). LDLR-A2 is involved in an extensive network of electrostatic and hydrogen-bonding interactions with TC helix α5 (Fig. 2b) including an additional salt bridge between TC2 residue Arg122 and Glu136 of LDLR-A2. The overall quality of electron density in LDLR-A2 is higher than that of LDLR-A1. This is especially apparent for the bound Ca^2+^ ion and Ca^2+^-coordinating residues and disulfide bond pairs. We also observe higher average B-factors for bound Ca^2+^ ions in LDLR-A1 compared with LDLR-A2 (∼80 Å^2^ and 44 Å^2^, respectively). Overall these results indicate increased motion/conformational disorder in LDLR-A1 compared with LDLR-A2.

### Dependence of TC-CD320 binding affinity on pH

**Figure 3:**
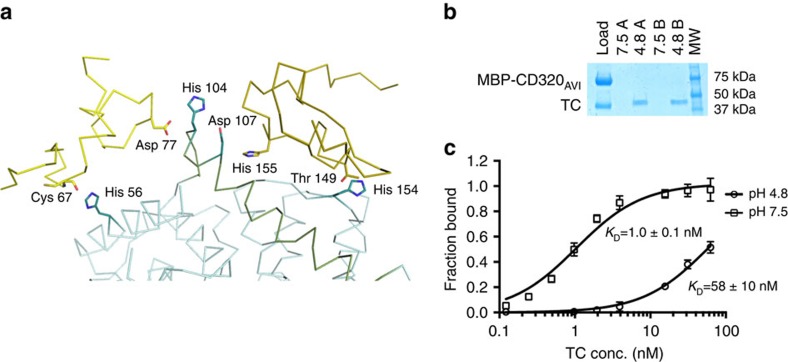
Interface histidines and pH dependence of TC-CD320 affinity. (**a**) Histidine residues identified at the TC-CD320 interface: CD320 (yellow and gold) and TC (blue) are shown as backbone traces, with histidine residues at the binding interface shown as respectively coloured sticks. (**b**) SDS–polyacrylamide gel electrophoresis (SDS–PAGE) showing TC dissociation from an immobilized CD320_AVI_-TC complex at pH 4.8 but not at pH 7.5. Two successive washes (A and B) are shown. (**c**) TC-CD320 binding curves at pH 4.8 and 7.5 obtained from solid-phase binding experiments (*n*=5, error bars indicate s.d.).

Ligand interactions of the LDLR family members are thought to be modulated by histidine residues located at the binding interfaces and responding to changes in pH, a mechanism termed the ‘histidine switch'^16^,^36^,^37^,^38^,^39^. An analysis of the TC-CD320 interface revealed three histidine residues from TC (His56, His104 and His154) and one from CD320 (His155) that may play a role in modulating the interaction (Fig. 3a). Among the observed interactions, the contact between His104 of TC and Asp77 of LDLR-A1 is notable because the backbone carbonyl of Asp77 coordinates the central Ca^2+^ ion. Furthermore, the backbone carbonyl of the interface residue His155 of CD320 also coordinates the central Ca^2+^ ion in LDLR-A2. This histidine also interacts with Asp157 of LDLR-A2, one of the acidic necklace forming residues. The above suggests that an intricate structural link exists between the pH-dependent interface and the Ca^2+^-coordination in the LDLR-A2 domain. Finally, His104 of TC is in a special location where it could interact with residues from both LDLR-A1 and LDLR-A2.

We analysed the effect of changes in pH on the TC-CD320 interaction by incubating an immobilized complex of TC and biotionylated receptor (CD320_AVI_) at pH 4.8 and pH 7.5 followed by SDS–polyacrylamide gel electrophoresis analysis. We observed a release of TC from the complex at low, but not at physiological pH (Fig. 3b). We quantitated this effect using our solid-phase binding assay by comparing binding of isolated tag-less TC to immobilized CD320_AVI_ at pH 4.8 and pH 7.5. As shown in Fig. 3c, the affinity of TC for CD320 is significantly reduced at lower pH (*K*_D_∼1.0 nM at pH 7.5 and∼58 nM at pH 4.8).

### Structure of the TC-CD320ΔE88 complex

Deletion of a single glutamate (ΔE88) in LDLR-A1 of CD320 has been associated with Cbl deficiency observed in patients carrying the mutation^40^. To investigate whether a structural effect was underpinning this defect, we generated a complex of TC with CD320ΔE88, characterized it biochemically and determined its structure at 2.6 Å resolution (Supplementary Fig. 3). Protein expression and purification were near identical to the wild-type complex. However, the purified complex displayed increased propensity to form non-specific multimers as analysed by SEC (Supplementary Fig. 4), requiring an additional SEC purification step for crystallization. As the mutant complex structure was of lower resolution than that of the wild type, we confirmed the positions of disulfide bonds and bound Ca^2+^ ions by using anomalous difference peaks from data collected at a wavelength of 1.77 Å.

The TC-CD320ΔE88 structure revealed a similar domain arrangement and binding interface as the wild-type protein and we observed no significant changes in TC or the LDLR-A2 domain, ruling out a gross structural effect of the ΔE88 mutation. As seen for the wild-type protein, the region of LDLR-A1 carrying the deletion (EEE loop) revealed poor electron density and higher B-factors when compared with that of LDLR-A2 (average B-factors for LDLR-A1 and A2 Ca^2+^ ions ∼112 and 56 Å^2^, respectively). Moreover, electron density for the Ca^2+^ binding residue Glu86 as well as Glu87 and disulfide forming Cys88 (equivalent to Cys89 in wild type) was missing. However, the centrally bound Ca^2+^ as well as the LDLR-A1 acidic necklace was clearly visible in our structure indicating intact Ca^2+^ binding. Even though the apparent Tm of the complex was slightly lower than that of the wild-type protein, approaching that of the LDRL-A2 domain alone (Supplementary Fig. 3b), the mutant protein bound TC with equivalent affinity to wild type with a calculated *K*_D_ of 1.5 nM (Supplementary Fig 3c).

### Role of the EGF domain of CD320 in TC binding

**Figure 4:**
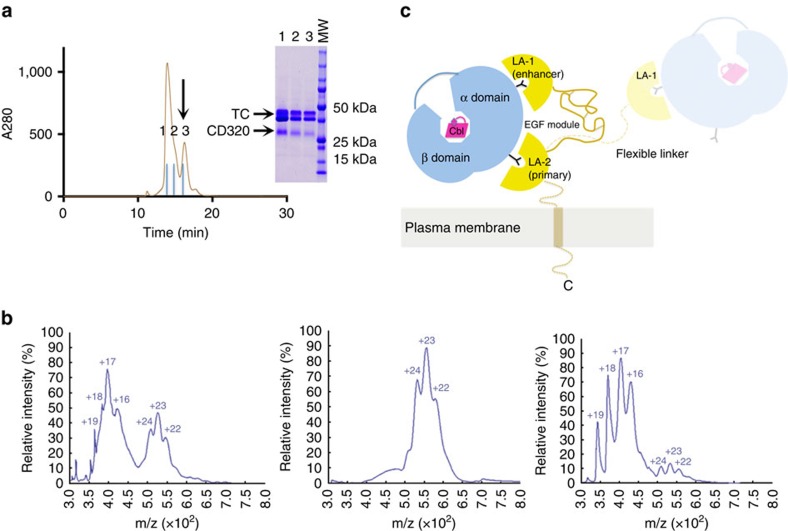
Role of EGF-like domain in TC-CD320 complex formation. (**a**) SEC profile of TC-CD320(Gly_4_Ser)_4_ construct, revealing higher mass peaks indicating multimeric species. Arrow indicates position of 1:1 TC-CD320 peak. Peak fractions indicated above SEC profile correspond to lanes in SDS–polyacrylamide gel electrophoresis (SDS–PAGE). Note that TC appears as a double band depending on polyacrylamide percentage and SDS–PAGE running conditions. (**b**) Native electrospray ionization mass spectrometry (ESI-MS) analysis of TC-CD320(Gly_4_Ser)_4_ complex. Each peak cluster comprises different charged species of a single macromolecular assembly formed due to the presence of residual salt from protein purification and correspond to approximate molecular weights (MWs) of 65 and 120 for the lower and higher molecular weight species, respectively (left panel). Analysis of Individually purified SEC peaks shows isolated high MW (middle) and low MW (right) species. (**c**) Model of properly oriented LDLR-A1 and LDLR-A2 domains (strong yellow) acting in tandem to specifically bind a single TC molecule. Dashed line indicates a flexible linker with proposed second TC molecule (faded yellow) binding.

The exact function of the EGF-like domain in CD320 has not yet been established and its deletion in CD320 was shown to have no effect on TC binding^8^. The EGF-like domain of CD320 is not visible in our electron density maps despite chemically being present, suggesting positional disorder in the crystal. To assess what effect the domain has on CD320 complex formation with TC, we generated a mutant of CD320 in which the EGF-like domain was replaced by (Gly_4_Ser)_4_ sequence, which constituted a linker long enough to offer unrestricted movement and flexibility to the LDLR-A modules that the disulfide-rich EGF-like domain would not allow. The mutant protein was co-purified with TC and appeared biochemically stable and capable of TC binding. However, we found that the resulting construct yielded additional species of higher molecular weight in SEC in addition to the 1:1 stoichiometry complex peak seen for wild type (Fig. 4a). As shown in Fig. 4b, these results were confirmed by native electrospray ionization mass spectrometry analysis of the TC-CD320(Gly_4_Ser)_4_ complex as well as its individually purified SEC peaks. We observed single species of ∼120 kDa mass for the larger SEC peak and 65 kDa mass for the smaller SEC peak, which we interpret as 2:1 or 2:2 TC:CD320 species and a single 1:1 complex, respectively.

## Discussion

**Figure 5:**
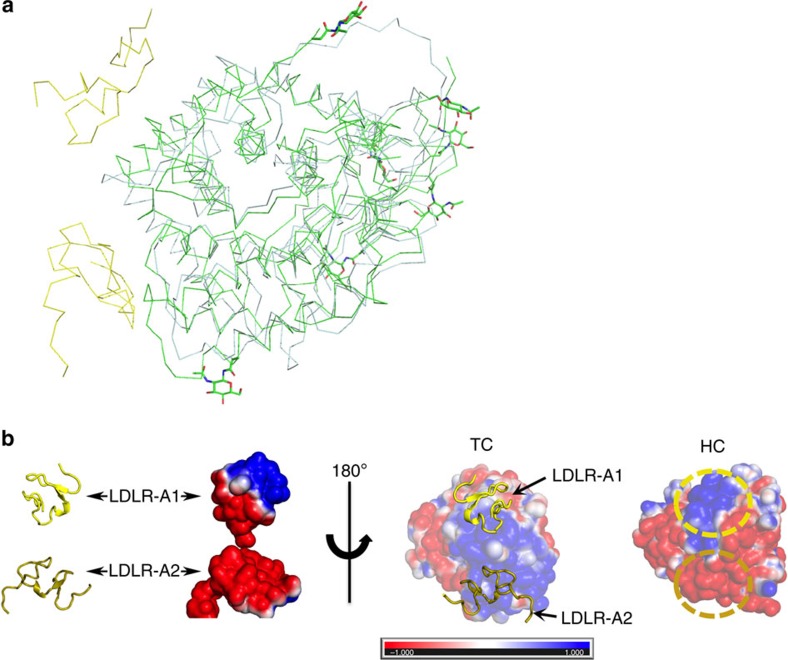
Comparison of CD320-bound TC with free HC (pdb ID 4KKI). (**a**) Superposition of HC with CD320-bound TC revealing position of glycosylated residues (green sticks) of HC. (**b**) Electrostatic potential maps of CD320 (2nd from left), TC (2nd from right) and HC (right) with the latter two rotated 180° along the *y* axis in relation to CD320 to reveal the receptor interaction surfaces. For clarity, CD320 LDLR-A1 (yellow) and LDLR-2 (gold) are also show in cartoon format. The regions of HC corresponding to equivalent TC binding regions for LDLR-A1 and LDLR-A2 are highlighted (dotted yellow and gold ovals, respectively).

Our structure offers a basis for the selectivity of CD320 for TC over the homologous and structurally similar HC. This is especially relevant in light of HC's suggested role as a scavenger protein for corrin-like substrates in the blood stream that could otherwise bind TC and enter the cell and interfere with downstream Cbl metabolism. Unlike TC, both IF and HC are glycoproteins and it was previously suggested that N-linked glycosylation of surface asparagines of HC might prevent its binding to CD320 (ref. 21). A comparison of receptor-bound TC with the recently determined HC structure (Fig. 5a) rules out such an explanation, as the glycans are not located at the appropriate surface. Rather, TC and HC have different electrostatic potentials at the relevant surfaces (Fig. 5b), with Lys105 and Lys114 in TC replaced by Asp92 and Asn102 in HC, respectively. The resultant electrostatic mismatch is especially pronounced for regions of HC that would contact LDLR-A2, which is the major determinant of stable complex formation. Thus, HC cannot bind CD320 due to electrostatic repulsion

The finding that the TC-CD320 interaction only involves the alpha domain of TC is in contrast to the recognition interface of IF with Cubilin, where both the α and β domains of holo-IF are involved^19^,^41^. Our findings suggest that in the absence of gross structural changes in the α domain on loss of bound Cbl, both holo and apo-TC should be able to interact with CD320. Earlier studies have indeed pointed to receptor recognition of apo-TC, although a higher affinity was reported for holo-TC (refs 42, 43, 44). The TC-CD320 binding interface is only slightly larger than that of the LDLR-RAP complex (buried surface area ∼760 Å^2^) and whereas the two LDLR-A modules in RAP contribute equally to the buried surface area and use virtually identical modes of ligand recognition, LDLR-A2 in CD320 serves as the primary determinant of high affinity binding. LDLR-A1, which displays low *in vitro* affinity to TC on its own, probably has a role in enhancing the specificity and affinity of CD320 for TC. The mutant CD320ΔE88, although more prone to forming non-specific complexes with TC (Supplementary Fig. 4), displays high thermal stability as well as tight TC binding. At least *in vitro*, these findings rule out a compromised ability of CD320ΔE88 to bind TC. Additional studies are needed to explain the *in vivo* effect of the mutation on receptor regulation and trafficking. Our data also support a structural role of the EGF-like domain of CD320 in influencing the flexibility and the orientation of the two LDLR-A domains to ensure that both LDLR-A domains bind to a single TC (Fig. 4c), thereby preventing formation of non-specific multimers that could potentially be detrimental to proper ligand internalization.

The combination of an acidic environment and decreased Ca^2+^ concentration has been implicated in the release of ligands of LDLRs during endocytosis (as recently reviewed^45^). However, no direct demonstration of pH-mediated dissociation of the TC-CD320 complex was reported. Our *in vitro* finding that the affinity of TC for CD320 is reduced more than 50-fold at low pH is in line with the expected receptor-ligand segregation and TC delivery to the lysosomal pathway. Our *in vitro* system sets the stage for addressing more detailed questions in future studies. These include whether acidification promotes Ca^2+^ release from the LDLR-A domains, how CD320 changes at low pHs, and whether such changes alter the ability of CD320 to bind potential regulators.

Finally, the ability to produce a stable and intact TC-CD320 complex, along with knowledge of the binding interface at atomic detail, might facilitate the design and manufacture of small molecule as well as immunotherapeutic regulators that could selectively disrupt the interaction in certain tumours overexpressing CD320. As for the latter, we have put in significant effort towards and been successful in the generation of camelid nanobodies against the TC-CD320 complex targeting various epitopes. These have the potential not only to aid in capturing novel conformational states of CD320 for structure determination, but also add to list of potential tools available for diagnostic or therapeutic applications in cancer treatments.

## Methods

### Protein expression and purification

The gene for human TC harbouring a R209Q mutation (GenBank ID BC001176.1) was obtained from ImaGenes (Life sciences). All CD320 constructs were N-terminally fused to a nona-histidine tagged MBP construct with a tobacco etch virus (TEV) protease cleavage sequence inserted between the MBP and receptor genes. The native secretion signals for both CD320 and TC constructs were replaced with a Melittin secretion signal for enhanced secreted expression in *Spodoptera frugiperda* (Sf21 cells) using the Multibac expression system^46^. CD320 and TC constructs were cloned into the dual expression pFL vector and bacmids, and viruses were generated according to recommended procedures. For CD320, residues 31–199 and 53–199 were used. LDLR-A1 and LDLR-A2 constructs comprised residues 53–95 and 125–199, respectively. The TCα construct comprised residues 1–290 of TC. Sf21 cells were grown and maintained in SF900-II medium and protein was expressed for 96 h post infection. Bulk medium was then harvested, supplemented with 1 μM Cbl-CN, and its pH was adjusted to 6.5 before being applied to Ni-NTA resin equilibrated in 20 mM HEPES pH 7.0, 0.5 mM CaCl_2_ and 150 mM NaCl. The resin was washed with 20 mM HEPES pH 7.0, 300 mM NaCl, and 40 mM imidazole and the protein eluted with 20 mM HEPES pH 7.0, 150 mM NaCl, 0.5 mM CaCl_2_ and 200 mM imidazole buffer. Eluted protein was desalted in 20 mM Tris pH 7.5, 0.5 mM CaCl_2_ and 150 mM NaCl (Buffer A) before overnight cleavage of the 9xHis-MBP module with His-tagged TEV protease (prepared in-house) at a w/w ratio of 1:10 (TEV:CD320). The resultant TC-CD320 complex displayed weak affinity for Ni^2+^-NTA and could be purified from excess receptor through a second Ni^2+^ affinity purification step with excess receptor separated in the flow through and the intact receptor eluting with 20 mM Imidazole. Final samples were desalted into Buffer A, concentrated to 10–20 mg ml^−1^and flash frozen in liquid N_2_. The flexible linker mutant of CD320 was generated by replacing residues 96–135 of CD320 with the eicosapeptide (Gly_4_Ser)_4_. Protein expression was performed as for wild-type TC-CD320. Where required, CD320 and its variants were individually expressed without TC in the same cloning/expression system.

Biotinylated receptor was prepared by adding a C-terminal Avi Tag (GLNDIFEAQKIEWHE) to CD320 and its variants. Expression and purification was identical to the wild-type complex except that no MBP cleavage was performed. Overnight biotinylation with the biotin ligase BirA (prepared in-house) was performed by adding 5–10 ug BirA per mg purified receptor in Buffer A containing 10 mM magnesium acetate, 10 mM ATP and 50 μM biotin. Biotinylation was confirmed by gel shift analysis on mixing desalted, biotinylated samples with streptavidin before SDS–polyacrylamide gel electrophoresis.

### TC binding assay

Tag-less TC was obtained by dissociating a complex of TC and biotinylated CD320 immobilized on Streptactin resin (IBA) with four column volumes of Buffer B (Buffer A lacking Ca^2+^ and containing 10 mM EGTA) at 37 °C for 30 min. The flow through containing TC was collected and the process repeated an additional three times. The pooled flow through samples were concentrated in an Amicon filter (30 kDa MWCO) and desalted in Buffer A. Protein concentrations were determined using a Bio-Rad DC Protein Assay Kit and A_280_ measurements.1–5 pMol receptor was added to each well of pre-blocked 96-well Neutravidin plates (ThermoFischer Scientific) for 1 h at room temperature (RT). All incubations were carried out on a plate shaker at 350 r.p.m. Unbound receptor was discarded and the wells were washed three times with 200 μl Buffer A containing 0.05% Tween-20 (Buffer AT). TC (100 ul) serially diluted in Buffer AT was added to the wells for 1 h at RT. Unbound TC was discarded and the wells washed five times with 200 μl Buffer AT. To analyse binding at different pHs, Buffer A was modified to contain 100 mM Tris-HCl pH 7.5 or 100 mM sodium acetate pH 4.8 instead of 20 mM Tris-HCl pH 7.5 and used for serially diluting TC. An additional wash step in TC dilution buffer was added to equilibrate the wells before TC binding as well as after discarding unbound TC. TC antibody A-5 (Santa Cruz Biotechnology, Inc, Catalogue # sc-137017) was diluted to 1 μg ml^−1^ (1:200) in Buffer AT and 100 μl was added to the wells for 30 min at RT. Unbound antibody was discarded and the wells washed three times with Buffer AT. Horse radish peroxidase (100 μl) conjugated Goat mouse Anti IGg (ThermoFischer Scientific, Catalogue # A16072) diluted to 1 μg ml^−1^ (1:1,500) in Buffer AT was added to the wells for 30 min. Unbound antibody was discarded and the wells washed three times with Buffer AT followed by development with the TMB Substrate Kit (ThermoFischer Scientific). Absorbance (450 nM) measurements were made with a BioTek Synergy HT plate reader. Absorbance readings were plotted against TC concentrations and curves fitted in GraphPad PRISM using a single-site specific binding equation, and normalized to the Calculated Bmax for direct comparison. All experiments were done in replicates of 5.

### Thermostability assays

Frozen protein samples were thawed and diluted to 0.5–1 mg ml^−1^ in Buffer A. A thermocycler (Bio-rad) was used to heat 80–100 ul samples at the selected temperatures for 10 min followed by rapid cooling to 4 °C before spinning down at 120,000*g* for 20 min. Samples were loaded onto a G3000Swxl (TOSOH Biosciences) column pre-equilibrated in Buffer A at 4 °C. Thermostability curves were plotted in GraphPad PRISM based on complex peak heights and fitted using a sigmoidal dose response (variable slope) equation. Protein used for assays in the presence of EGTA was diluted 20 × to 0.5–1 mg ml^−1^ in Buffer B, which was also used for SEC.

### Chemical Cross-linking

The protein complexes at 10 mg ml^−1^ were mixed with a 10% glutaraldehyde solution in a 10/1 (v/v) ratio for one hour at RT in buffer solutions. The mixture was further diluted with the original protein buffer solution or water before mass spectrometric analysis.

### Mass spectrometry

A commercial MALDI-TOF/TOF mass spectrometer (model 4800 plus, AB Sciex, Darmstadt, Germany) equipped with a high-mass detector (HM2, CovalX AG, Zurich, Switzerland) was used. All measurements were performed in the linear positive ion mode with standard settings. Ionization was achieved with an Nd:YAG laser (355 nm) with the energy just above the threshold for ion formation. Each mass spectrum was the average of 1,000 laser shots acquired at random sample positions without searching for hot spot. Sinapinic acid (20 mg ml^−1^ in water/acetonitrile/TFA, 49.95/49.95/0.1, v/v/v) was used as the matrix. Matrix (0.5 μl) was first spotted on a stainless steel plate and allowed to dry under ambient conditions. Afterwards, 0.5 μl of the sample was spotted on the top of the matrix and also dried under ambient conditions. In the final step, 0.5 μl of the matrix was spotted. All mass spectra were baseline corrected and smoothed using a Savitzky-Golay algorithm by Igor Pro 6.2, WaveMetrics, Oregon, USA.

Native ESI-mass spectrometry was carried out on a commercial hybrid quadrupole/time-of-flight mass spectrometer Synapt G2-S equipped with a Z-spray interface followed by the StepWave ion guides (Waters, Manchester, UK). The instrument was operated in the Resolution mode (resolution>20,000) in positive ion mode. For calibration, CsI clusters (10 mg ml^−1^) dissolved in 50% (v) isopropanol was used. The recorded spectra were averaged (500 scans), smoothed with a moving algorithm (width of three steps) and centroid spectra were generated at 80% peak height. After polynomial fitting, the mass axis was calibrated. Typically, 3–5 μl of sample were loaded into 1 μm Au/Pd coated glass needles (Thermo Scientific, Madison, WI, USA). The source temperature was kept at 30 °C. The backing pressure was set to 0.5 bar to assist sample flow. The capillary voltage was set to 0.95 V to generate the electrospray. The sampling cone voltage and the source offset were optimized to 150 and 50 V respectively, to ensure efficient ion transmission. Further, to assure better declustering of salt adducts, the trap collision energy and the transfer collision energy were kept at 30 and 15 V, respectively. The quadrupole transmission range was adjusted for the desired m/z range. The mass spectra were recorded in a m/z window of 50–8,000 with a scan time of 2 s and an interscan delay of 0.1 s. Each native electrospray ionization mass spectrometry spectrum represents the combination of 150 individual scans. Mass spectra were acquired using Mass Lynx software (version 4.1, Waters, Manchester, UK). All mass spectra were baseline corrected, normalized and smoothed using MATLAB_R2014a.

### Crystallization and structure determination

For crystallization of the wild-type TC-CD320 complex, frozen samples were thawed and diluted to 10 mg ml^−1^ and mixed with well buffer (9.6% PEG8000, 160 mM Ca(OAc)_2_, 80 mM MES pH 6.2, and 20 mM MgCl_2_) in a 1:1 ratio at 20 °C in sitting drop plates. Crystals were cryo-protected by exchanging the drop solution with 9.6% PEG8000, 160 mM Ca(OAc)_2_, 80 mM MES pH 6.2, and 2 0 mM MgCl_2_ 20 mM Tris pH 7.5, 150 mM NaCl, 0.5 mM CaCl_2_, 1 μM Cbl-CN and 25% Glycerol and flash frozen in liquid Nitrogen. An N-terminal 22 amino acid deletion of the receptor led to higher quality crystals that were used for the final structure determination.

Crystals of the ΔE88 mutant were obtained by purifying the complex by SEC in Buffer A before concentrating to 10 mg ml^−1^ and mixing with well buffer (180 mM L-Proline, 11.7% PEG3350, 90 mM Hepes pH 7.5, and 30 mM Glycyl-Glycyl-Glycine) in a 2:1 ratio at 20 °C in sitting drop plates. Crystals were cryo-protected by exchanging the drop solution with Cryo-buffer (108 mM L-Proline, 54 mM Hepes pH 7.5, 7.2% PEG3350, 0.6 mM CaCl_2_, 24 mM Tris pH 7.5, 180 mM NaCl, 1 μM Cbl-CN and 25% Glycerol) and flash frozen in liquid Nitrogen. Data were collected at the X06SA microfocus beamline (Swiss Light Source) at a wavelength of 1 Å. A complete data set from a single wild-type crystal was collected in six discreet wedges and processed using denzo and scalepack (HKL Research). The structure was determined by molecular replacement using PHASER in PHENIX (ref. 47) using a TC monomer from the Cbl-H_2_0 bound human TC structure (PDB 2BB5) as a search model. Model building and refinement were carried out using COOT (ref. 48) and PHENIX. Anomalous data sets from an identical crystal collected at 1.6 Å wavelength was processed by denzo and scalepack. Two molecules of the TC-CD320 complex/asymmetric unit were found, molecule 1 comprising chains A and C and molecule 2 comprising chains B and D.

A complete data set for the ΔE88 mutant was processed by XDS (ref. 49) and aimless in CCP4 (ref. 50). The wild-type TC-CD320 structure was used as a search model for molecular replacement using PHASER in PHENIX. Model building and refinement were carried out using COOT and PHENIX. An anomalous data set collected at 1.77 Å wavelength was processed by XDS and aimless in CCP4. TC-CD320 complex molecule 2 was used to generate all figures using the PyMOL Molecular Graphics System (The PyMOL Molecular Graphics System, Version 1.8 Schrödinger, LLC). Part of the 2fo-fc electron density maps for both the wild-type and CD320ΔE88 complexes with TC are shown in Supplementary Fig. 6.

### Data availability

Atomic structure factors and coordinates for the TC-CD320 and TC-CD320ΔE88 structures have been deposited under accession codes 4ZRP and 4ZRQ, respectively. The authors declare that all relevant data sets used in this study are available on request.

## Additional information

**How to cite this article**: Alam, A. *et al.* Structural Basis of Transcobalamin Recognition by human CD320 Receptor. *Nat. Commun.* 7:12100 doi: 10.1038/ncomms12100 (2016).

## Supplementary Material

Supplementary Information Supplementary Figures 1-6.

## Figures and Tables

**Table 1:** Data collection and refinement statistics.

**Crystal:**	**TC-CD320**	**TC-CD320ΔE88**	**TC-CD320**_**ano**_	**TC-CD320ΔE88**_**ano**_
*Data collection*				
Wavelength (Å)	1.0	1.0	1.6	1.77
Space group	P43212	P43212	P43212	P43212
Cell dimensions				
*a*,*b*, *c* (Å)	98.10, 98.10, 356.0	98.40, 98.40, 356.3	97.94, 97.94, 356.5	97.81, 97.81, 354.7
*α,β,γ* (°)	90, 90, 90	90, 90, 90	90, 90, 90	90, 90, 90
Resolution (Å)	2.1	2.6	3.0	3.0
*R*_merge_	0.1(0.9)*	0.121(1.62)	0.11 (0.44)	0.186(0.97)
*I*/σ*I*	12(1.0)	16.8(2.2)	19.8 (7.2)	25.4 (6.1)
Completeness (%)	95.8(62.3)	99.9(100)	98.3 (97.4)	98.4 (97.1)
Redundancy	4.8(2.5)	10.8(11.2)	9.0 (9.3)	35.7 (37.0)
				
*Refinement*				
Resolution (Å)	2.1	2.6	–	–
No. reflections	101389	54979	–	–
*R*_work/_*R*_free_	0.1840/0.2075	0.2001/0.2305	–	–
No. atoms			–	–
Protein/Cbl	7515	7488	–	–
Ca^2+^ ions	6	4	–	–
Water	444	44	–	–
B-factors				
Protein/Cbl	58.6	74.4	–	–
Ca^2+^ ions	61.9	86.1	–	–
Water	55.0	62.4	–	–
r.m.s deviations				
Bond lengths (Å)	0.01	0.01	–	–
Bond angles (°)	1.93	1.89	–	–
Ramachandran plot			–	–
Favoured (%)	96.5	96.6	–	–
Allowed (%)	3.4	3.1	–	–
Disallowed (%)	0.1	0.3	–	–

^*^Values in parentheses are for the highest-resolution shell.
