# Supplementary material for: Structural basis of transcobalamin recognition by human CD320 receptor
Source: Nat Commun. 2016 Jul 14;7:12100. doi: 10.1038/ncomms12100 (PMC4947154; doi:10.1038/ncomms12100)
Supplement: Supplementary Information — Supplementary Figures 1-6. [file ncomms12100-s1.pdf]

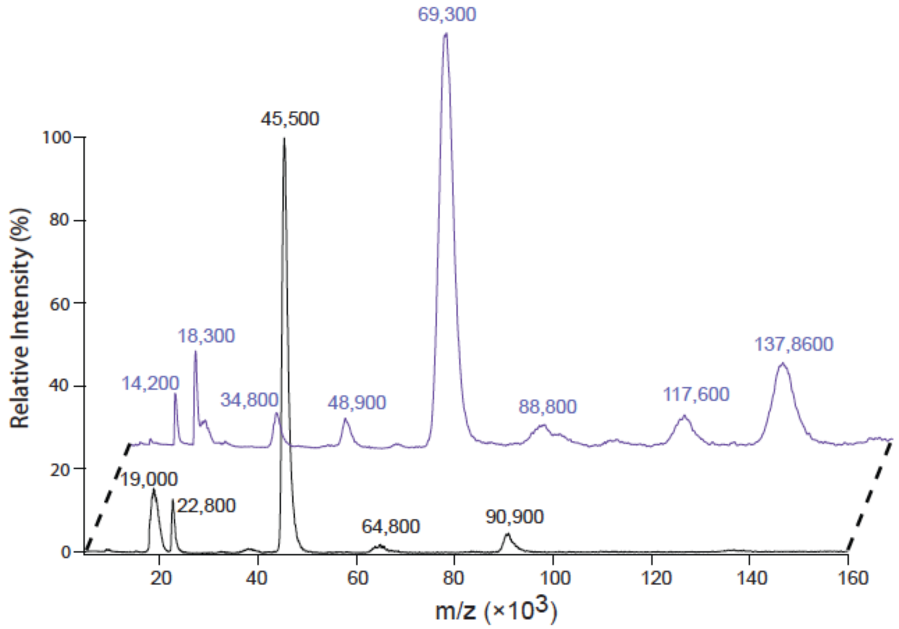

Supplementary Figure 1 Mass spectrometric analysis of the crystallized construct of TC-CD320 in the absence (black) and presence (blue) of cross-linker, revealing a predominant 1:1 complex peak upon cross-linking. The calculated molecular masses are indicated above the peaks. The theoretical masses of TC and the crystallized construct of CD320 (de-glycosylated) are 45.8 and 15.7 kDa, respectively.

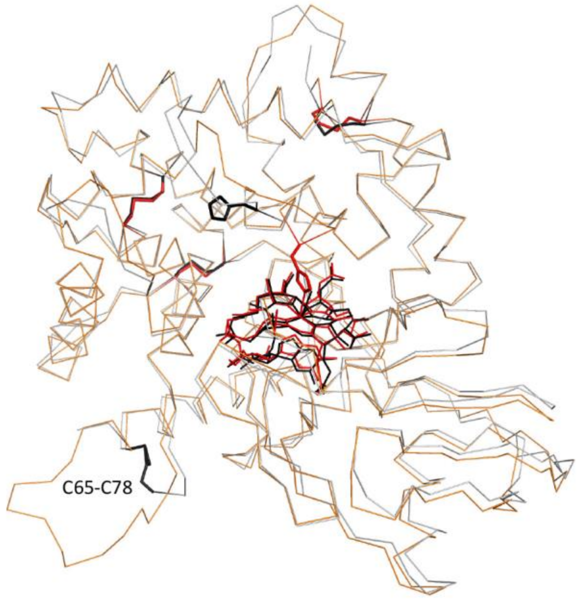

Supplementary Figure 2 Superposition of Cbl-bound TC from TC-CD320 complex structure (grey backbone trace and black sticks) with isolated TC (PDB 2BB5, orange) bound to Cbl-H_20 (red sticks). Disulfide linkages and His 173 for the two structures are indicated as black and red sticks, respectively with the Cys65-Cys78 linkage (missing in 2BB5) labeled.

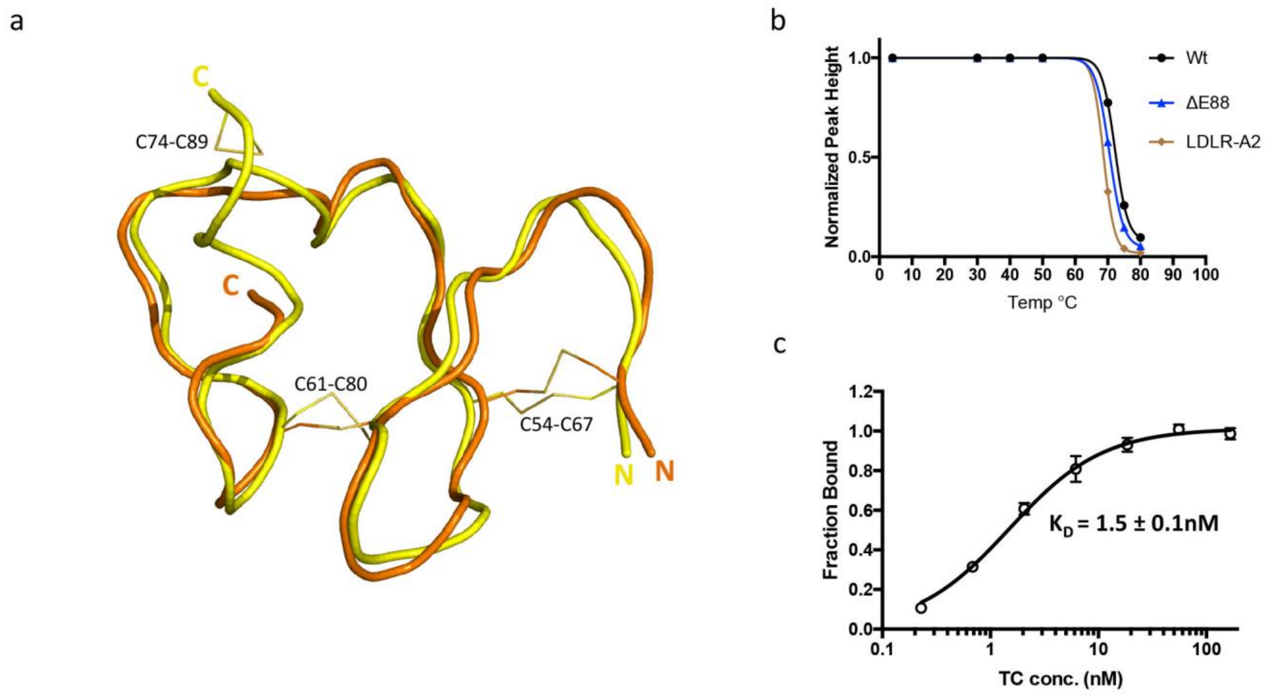

Supplementary Figure 3 Structural and biochemical characterization of TC–CD320(ΔE88). (**a**) Superposition of the LDLR-A1 domains of wild type TC-CD320 (yellow) and TC-CD320ΔE88 (orange), with disulfide pairs indicated by sticks and labeled. The Cys74-Cys89 disulfide pair is missing in the TC-CD320ΔE88 structure (**b**) Thermostability analysis showing in order of decreased stability the complexes of TC with WT CD320 (black), CD320ΔE88 (blue), and the isolated LDLR-A2 (brown). (**c**) Solid phase binding assay revealing high affinity binding of CD320 and TC (n=5, error bars indicate SD).

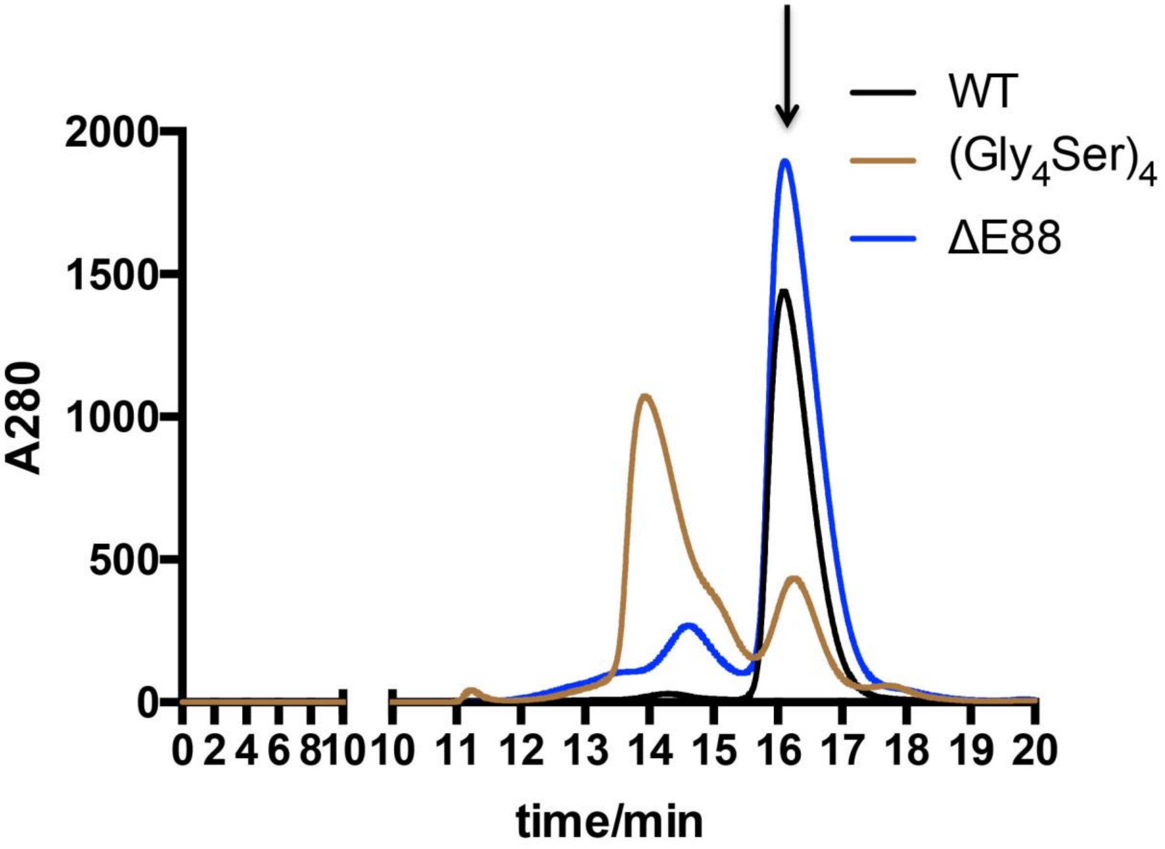

Supplementary Figure 4 SEC elution profiles of wild type (WT) and mutant TC–CD320 complexes displaying 1:1 TC–CD320 complexes (16-17 min. elution time) for the WT (black trace) and mutant CD320 constructs. Higher molecular weight peaks appear at 13-15 min elution time for the CD320 variants containing the $(Gly_4Ser)_4$ linker or the ΔE88 mutation (blue and brown traces, respectively). Arrow indicates elution peaks for 1:1 TC-CD320 complex (see figure 5a).

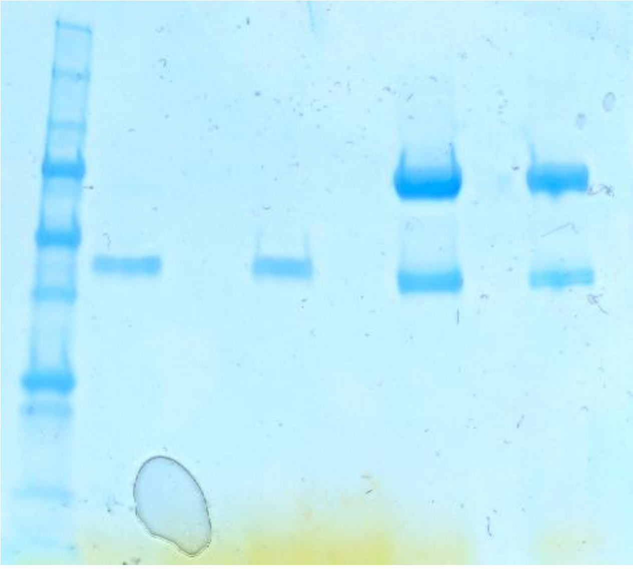

Supplementary Figure 5 Full SDS-PAGE gel from Fig 3b.

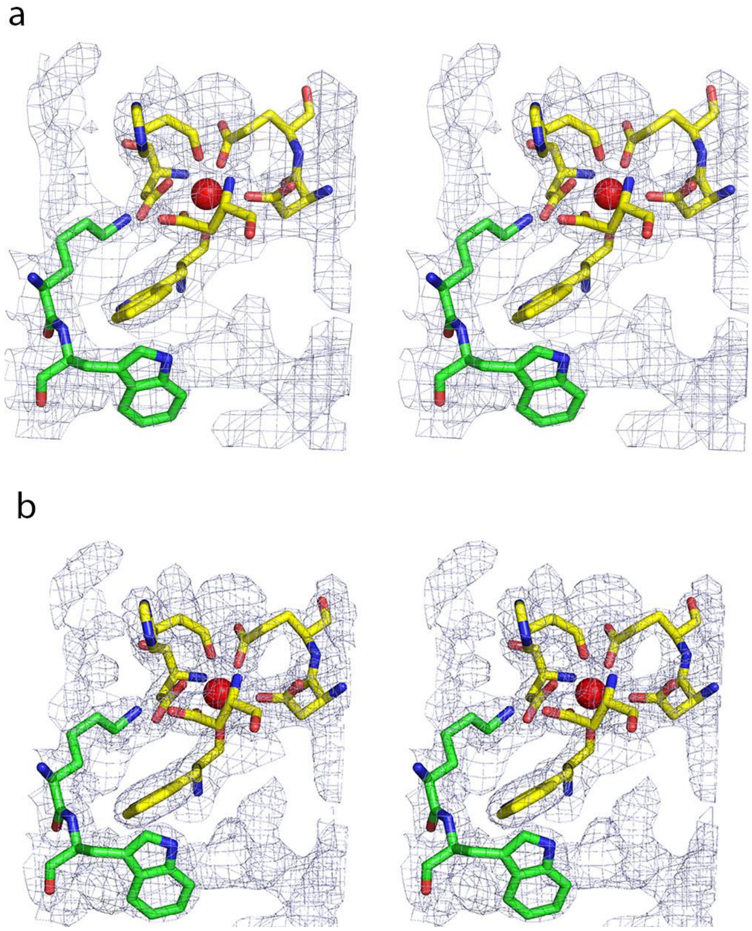

Supplementary Figure 6 Electron density maps for TC-CD320 and TC-CD320 ΔE88. (**a)** 2fo-fc electron density map for TC-CD320 complex centered on part of the LDLR-A2 (yellow) and TC (green) interaction interface contoured at 2.5σ. (**b**) 2fo-fc electron density map for TC-CD320 ΔE88 complex centered on the LDLR-A2 (yellow) and TC green) interaction interface contoured at 1.5 σ.
